# Clinical effects of erythritol, glycine and trehalose as subgingival air-polishing powders on non-surgical periodontal treatment:a systematic review with meta-analysis of randomized controlled trials

**DOI:** 10.3389/fphys.2025.1593204

**Published:** 2025-07-28

**Authors:** Yuan Zi-le, Tang Yan-xi, Zhou Ying, Wenjie Li, Chen Jun, Meilu Zhou

**Affiliations:** ^1^Department of Periodontology and Oral Mucosa, Changsha Stomatological Hospital, School of Dental Medicine, Hunan University of Chinese Medicine, Changsha, Hunan, China; ^2^Hunan Key Laboratory of Oral Health Research and Hunan 3D Printing Engineering Research Center of Oral Care and Hunan, Clinical Research Center of Oral Major Diseases and Oral Health and Xiangya School of Stomatology, Central South University, Changsha, China; ^3^Department of Orthodontics, Xiangya Stomatological Hospital, Central South University, Changsha, China; ^4^Department of Periodontics, Xiangya Stomatological Hospital, Central South University, Changsha, China

**Keywords:** subgingival air-polishing, non-surgical periodontal treatment, erythritol, glycine, trehalose

## Abstract

**Purpose:**

This study aimed to systematically compare the efficacy of erythritol, glycine, and trehalose as subgingival subgingival air polishing powders in non-surgical periodontal treatment (NSPT).

**Methods:**

Randomized controlled trials (RCTs) that met the inclusion and exclusion criteria were selected from PubMed, Embase, Web of Science, and Cochrane Library databases (up to August 2024). The sample size, treatment time, and outcome indicators including periodontal probing depth (PPD), clinical attachment level (CAL), and bleeding on probing (BOP) were extracted from the articles. Direct meta-analysis and network meta-analysis were performed by using “*R*”.

**Results:**

Nine RCTs met the inclusion and exclusion criteria. A total of 462 patients were included in the study. For PPD, the network meta-analysis showed that there was no statistical significance in the cross-comparison of erythritol, glycine and trehalose. However, erythritol (SUCRA = 84.1) has an advantage over trehalose (SUCRA = 48.0) and glycine (SUCRA = 28.5) in reducing PPD. The results of direct meta-analysis showed that there was no significant statistical difference in the improvement of outcome indicators such as PPD, CAL and BOP by the three subgingival polishing powders.

**Conclusion:**

The recommended order of priority for the use of three subgingival subgingival air polishing powders is as follows: erythritol, trehalose, and glycine.

**Strenaths and limitaions of this study:**

We recommend the priority for the use of the three powders was as follows: erythritol, trehalose, and glycine.Limitations:limited number of RCTs made it difficult to draw a test for publication bias.

**Systematic Review Registration:**

identifier CRD42022366792.

## 1 Background

Periodontitis is a common disease with a prevalence rate of 11% worldwide. Severe periodontitis may even lead to tooth loss, thereby reducing the quality of life (TaeHyun Kwon and Liran, 2021). The treatment of periodontal disease requires multiple courses of treatment and long-term oral hygiene maintenance of the patient (Sanz et al., 2020). Removal of subgingival biofilm and dental calculus is the basis for the treatment of periodontal disease (Alex Nogueira Haas et al., 2021). Probing pocket depth (PPD), clinical attachment level (CAL),and bleeding on probing (BOP) are commonly used to evaluate the therapeutic effect of periodontal disease (Alex Nogueira Haas et al., 2021; Loos and Needleman, 2020; Tonetti et al., 2018). In recent years, with the development of research, non-surgical treatment, including subgingival air polishing and the use of ultrasonic instruments, has become a new treatment method for periodontal disease (Alexia Vinel et al., 2022). Among them, subgingival air polishing is the use of pressurized air, water, and different sandblasting powders to disrupt the overlying and subgingival biofilms. Glycine, erythritol and trehalose are commonly used subgingival air polishing powders.

Glycine, an amino acid and immunomodulator for gingival inflammation, is biocompatible and highly soluble, effectively removing subgingival biofilm and plaque. With a particle size of 25–65μm, it reduces discomfort during periodontal treatment and inhibits bacteria, but promotes inflammation and delays wound healing *in vitro*. Trehalose, a non - cariogenic disaccharide, has a similar grinding effect on dental materials as glycine, with a particle size of 25–35 μm. It can reduce bacterial load but is less antibacterial than glycine, and has no significant impact on wound healing. Erythritol, water - soluble and chemically stable, is used as a food additive. With a particle size of 14μm, it is less abrasive than glycine, and can remove biofilm and exhibit antibacterial effects in periodontal treatment. Both erythritol and glycine air - polishing powders have no adverse events. However, currently there are no studies comparing the therapeutic effects (PPD, CAL, BOP) of these three types of subgingival abrasive powders. In this study, a meta-analysis was conducted to compare the efficacy of these three air-polishing powders, aiming to provide a theoretical basis for clinical decision-making.

## 2 Materials and methods

A protocol has been registered at the International Prospective Register of Systematic Reviews (No. CRD42022366792). The content of this article is consistent with the protocol.

1. Criteria for Considering Studies for this Review.

Only English-language RCTs with follow-up of at least 3 months were considered for inclusion and were organized by the PICO (patient, intervention, comparison, outcomes) method, according to the following points:

•Participants: Participants diagnosed with periodontitis were considered. Periodontitis was defined as the 2018 EFP/AAP periodontitis case classification.
•Interventions: The considered subgingival air polishing powders for NSPT include trehalose, erythritol and glycine.
•Outcomes: The following outcome indicators were considered as primary outcome measures: PPD; CAL and BOP.
•Risk-of-Bias Assessment: ROB2.0.
•Software for Statistical Analysis: R-Project 4.0.0.

2. Exclusion Criteria for Considering Studies for this Review.

• *In vitro* studies or animal research
• Systematic or narrative reviews, case reports, case series or letters to the editor
• Research on implants
• Experiments with gingivitis, not periodontitis
• Study on non-use of subgingival air polishing powder and subgingival air polishing equipment during NSPT
• Patients who had received periodontal therapy within 6 months
• Insufficient/missing/unpublished data
• Studies that did not report the primary outcome indicators (PPD, CAL, BOP).

Detailed search strategies were used to identify RCTs included in this meta-analysis. Search strategies for the Embase, Web of Science, PubMed, and Cochrane databases were presented in Table 1 and were updated on August 2024.

**TABLE 1:** Electronic databases and search strategies.

Database	Search strategy
Embase	#1 (‘air polishing’/exp) #2 (‘air powder polishing’:ab,ti) #3 (‘subgingival polishing’:ab,ti) #4 (#1 OR #2 OR #3) #5 (‘periodontal treatment’:ab,ti) #6 (‘periodontal therapy’:ab,ti) #7 (‘subgingival scaling’:ab,ti) #8 (#5 OR #6 OR #7) #9 (#4 AND #8)
PubMed	((((((“Periodontal Treatment/Therapy” [Mesh] OR “Subgingival Scaling” [Mesh]) OR (periodontal treatment)) OR (periodontal therapy)) OR (Subgingival Scaling)) AND (((((“Air Polishing” [Mesh] OR “Air Powder polishing” [Mesh] OR “Subgingival Polishing “ [Mesh]) OR (“Air Polishing”)) OR (“Air Powder Polishing”))) OR (“Subgingival Polishing “)
Cochrane	#1 MeSH descriptor: [air polishing] explode all trees #2 (air powder polishing):ti,ab,kw #3 (subgingival polishing):ti,ab,kw #4 (#1 OR #2 OR #3) #5 (periodontal treatment):ti,ab,kw #6 (periodontal therapy):ti,ab,kw #7 (subgingival scaling):ti,ab,kw #8 (#5 OR #6 OR #7) #9 (#4 AND #8)
Web of Science	#1 ((ALL= (air polishing)) OR ALL=(air powder polishing)) OR ALL=(subgingival polishing) #2 ((ALL= (periodontal treatment)) OR ALL=(periodontal therapy)) OR ALL=(subgingival scaling) #3 (#1 AND #2)

Note. ab = abstract; ti = title; kw = keyword; Mesh = Medical Subject Headings.

The search strategies were designed to identify relevant studies on the use of air polishing in periodontal treatment across different databases.

The identification and selection of studies was performed by two independent investigators (Y.Z. and Y.T.) who screened the titles, abstracts, and full texts of the articles. Disagreement between the two reviewers was solved by discussion with the attendance of another author (J.C.). The data were extracted independently and entered into a computer by two review authors (Y.Z. and Y.T.) using specifically designed data collection forms. Patient characteristics, treatment methods, clinical outcomes, and study quality were systematically documented. When clinical data on one or more of the outcome variables were not published/reported in the original article, the authors of the RCT in question were contacted and kindly asked to send their raw (unpublished) data for inclusion in the statistical model. In case of missing data or if the authors did not answer, RCTs were considered ineligible for inclusion in the present meta-analysis.

## 3 Results

### 3.1 Search results

**FIGURE 1:**
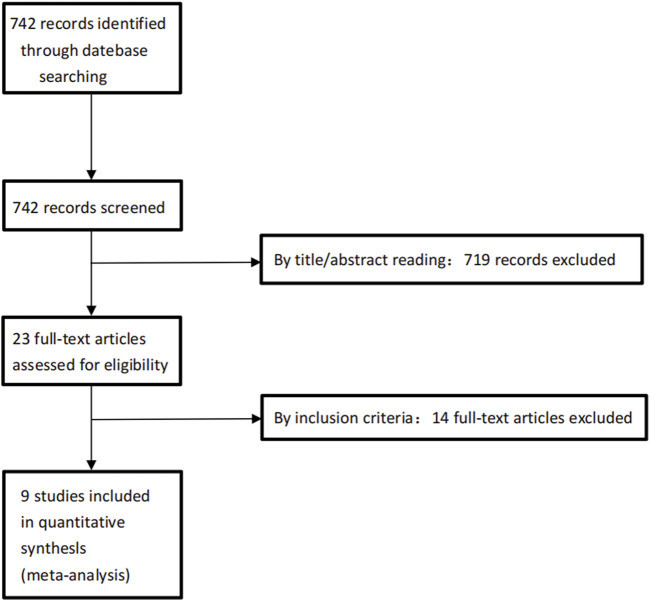
Flow diagram in PRISMA (Preferred Reporting Items for Systematic Reviews and Meta-Analyses) format of the screening and selection process.

**FIGURE 2:**
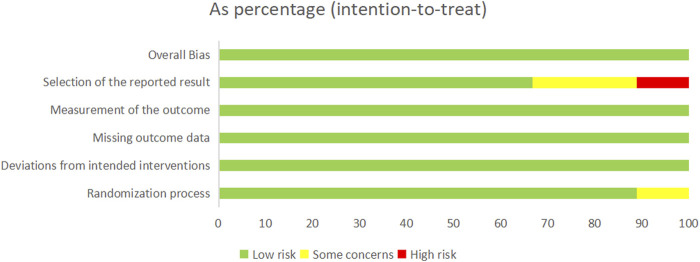
Risk of bias graph.

The Embase, PubMed, Web of Science and Cochrane Library databases yielded results for 149, 177,228 and 188 articles published, respectively. Nine articles were included after full-text reading (Table 1). Detailed steps were presented in Figure 1. In these nine articles, a total of 497 patients were followed up for 12 months, and all of which were RCTs. The characteristics of these nine articles were presented in Table 2. The results of the literature quality evaluation (based on the Cochrane handbook) were summarized in Figure 2.

**TABLE 2:** Characteristics of included studies.

Study	Study design,follow-up time	Patients and controls		Intervention VS comparison	Periodontal status	Location	Outcomes
		Before	After				
2020.Holger F. R. Jentsch	RCT,12 months	24 patients, 25 controls	21 patients, 21 controls	erythritol + SRP vs. SRP	stage II–III, grade B of the new classifcation scheme for periodontal and peri-implant diseases and conditions	Germany	PD CAL BOP
2018.Eon-Jeong Park	RCT,12 months	21 patients, 21 controls	21 patients, 21 controls	erythritol + SRP vs. SRP	moderate chronic periodontitis	Korea	PD CAL BOP
2022.Tihana Divnic-Resnik	RCT,12 months	21 patients, 21 controls	20 patients, 20 controls	erythritol + SRP vs. SRP	stage II–III, grade B periodontitis	Australia	PD CAL BOP
2013.Tobias T Hägi	RCT,12 months	20 patients, 20 controls	20 patients, 19 controls	erythritol vs. SRP	moderate to advanced chronic periodontitis	Switzerland	PD CAL BOP
2020.Anne B Kruse	RCT,12 months	52 patients, 52 controls	44 patients, 44 controls	trehalose vs. SRP	chronic periodontitis patients	Germany	PD CAL
2022.Anne B Kruse	RCT,12 months	53 patients, 53 controls	44 patients, 44 controls	trehalose vs. SRP	periodontitis grade A or B	Germany	PD CAL
2012.Thomas F Flemmig	RCT,12 months	15 patients, 15 controls	15 patients, 15 controls	glycine vs. SRP	chronic periodontitis patients	USA	PD BOP
2021.Wenyi Zhang	RCT,12 months	16 patients, 14 controls	12 patients, 12 controls	glycine + SRP vs. SRP	ten or more teeth with at least one site bleeding on probing depth of ≥ 5 mm	China	BOP
2018.Y C Tsang	RCT,12 months	27 patients, 27 controls	27 patients, 27 controls	glycine + SRP vs. SRP	having at least 20 teeth present excluding third molars; the presence of at least 2 teeth, with probing depth ≥5 mm and clinical attachment loss of ≥5 mm, in 2 quadrants; and radiographic signs of alveolar bone loss	China	PD CAL

Note. PPD, probing pocket depth; CAL, clinical attachment level; BOP, bleeding on probing; RCT, randomized controlled trial; SRP, Scaling and Root Planing. Data on 2020.Holger F. R. Jentsch adapted from “Adjunctive air-polishing with erythritol in nonsurgical periodontal therapy: a randomized clinical trial,” by Holger F R Jentsch 1 CF, Benjamin Kette 2, Sigrun Eick, 2020, *Bmc Oral Health*. Data on 2018.Eon-Jeong Park adapted from “Clinical and microbiological effects of the supplementary use of an erythritol powder air-polishing device in non-surgical periodontal therapy: a randomized clinical trial.,”by Park E-J, Kwon E-Y, Kim H-J, 2018, *Journal of Periodontal & Implant Science,* 48 (5). Data on 2022.Tihana Divnic-Resnik adapted from “The efficacy of the adjunct use of subgingival air-polishing therapy with erythritol powder compared to conventional debridement alone during initial non-surgical periodontal therapy. “ by Divnic-Resnik T, Pradhan H, Spahr A, 2022, *Journal of Clinical Periodontology,* 49 (6):547-555. Data on 2013.Tobias T Hägi adapted from “ Clinical outcomes following subgingival application of a novel erythritol powder by means of subgingival air polishing in supportive periodontal therapy: a randomized, controlled clinical study. “ by Tobias T Hägi PH, Giovanni E Salvi, Christoph A Ramseier, 2013, *Quintessence International*. Data on 2020.Anne B Kruse adapted from “ Effects of subgingival air-polishing with trehalose powder on oral biofilm during periodontal maintenance therapy: a randomized-controlled pilot study. “ by Kruse AB, Maamar R, Akakpo DL, 2020; *BMC, oral health,* 20 (1). Data on 2022.Anne B Kruse adapted from “ Subgingival subgingival air polishing with trehalose powder during supportive periodontal therapy: use of a conical shaped tip during a randomized clinical trial.” by Kruse AB, Wölki BJ, Woelber JP, 2022; *BMC, oral health,* 22 (1). Data on 2012.Thomas F Flemmig adapted from “ Randomized Controlled Trial Assessing Efficacy and Safety of Glycine Powder Subgingival air polishing in Moderate-to-Deep Periodontal Pockets. “ by Flemmig TF, Arushanov D, Daubert D, 2012, *Journal of Periodontology,* 83 (4):444-452. Data on 2021.Wenyi Zhang adapted from “ Clinical, inflammatory and microbiological outcomes of full-mouth scaling with adjunctive glycine powder air-polishing: A randomized trial. “ by Zhang W, Wang W, Chu C, 2021,*Journal of Clinical Periodontology,* 48 (3):389-399. Data on 2018.Y C Tsang adapted from “ Tsang YC, Corbet EF, Jin LJ., Subgingival glycine powder air-polishing as an additional approach to nonsurgical periodontal therapy in subjects with untreated chronic periodontitis. “ by Tsang YC, Corbet EF, Jin LJ, 2018, *Journal of Periodontal Research,* 53 (3):440-445.

The table presents the characteristics of studies included in the review, focusing on study design, interventions, outcomes, and comparisons.

## 4 Analysis results

According to the differences between the experimental group and the control group of the included RCT studies, the RCTs were divided into two categories for meta-analysis. The first category of RCTs was SRP + subgingival air polishing (experimental group) vs. SRP (control group), which allowed direct meta-analysis comparison between glycine and erythritol. The second category of RCTs was subgingival air polishing (experimental group) vs. SRP (control group), which allowed direct meta-analysis and network meta-analysis comparison among glycine, erythritol and trehalose. The result of the analysis include: the continuous outcome variables (PPD, CAL, BOP).

### 4.1 The experimental group received both SRP and subgingival air polishing, while the control group only received SRP

#### 4.1.1 PPD

**FIGURE 3:**
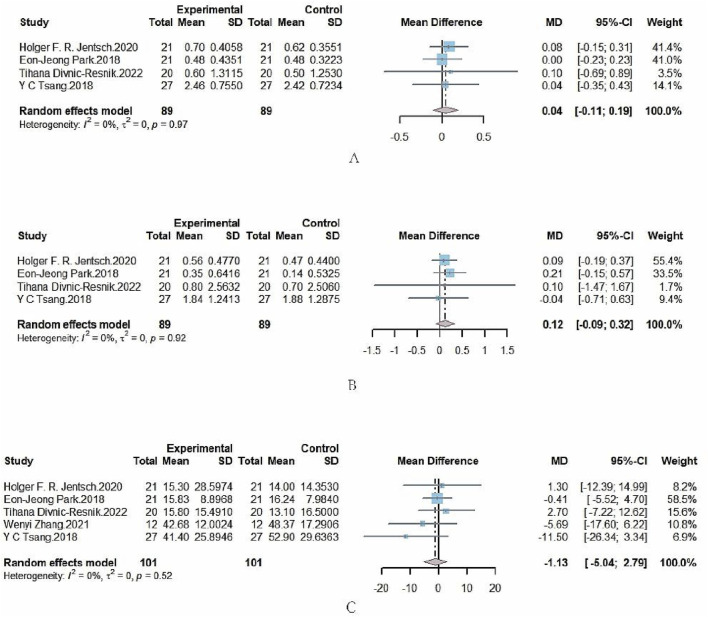
**(A)** Forest plot comparing PPD with SRP + erythritol versus SRP + glycine. **(B)** Forest plot comparing CAL with SRP + erythritol versus SRP + glycine. **(C)** Forest plot comparing BOP with SRP + erythritol versus SRP + glycine.

The forest plots (Figure 3A) describe mean difference (MD) and 95% credible intervals (Crl) of the comparison between interventions erythritol and glycine. Because the credible interval intersected the invalid line, the PPD difference between erythritol and glycine was not statistically significant with no statistical heterogeneity (I^2^ = 0, P = 0.97).

#### 4.1.2 CAL

As shown in Figure 3B, the CAL difference between erythritol and glycine was not statistically significant with no statistical heterogeneity (I^2^ = 0, P = 0.92).

#### 4.1.3 BOP

As shown in Figure 3C, the BOP difference between erythritol and glycine was not statistically significant with no statistical heterogeneity (I^2^ = 0, P = 0.52).

### 4.2 The experimental group only received subgingival air polishing, while the control group received SRP

#### 4.2.1 PPD

**FIGURE 4:**
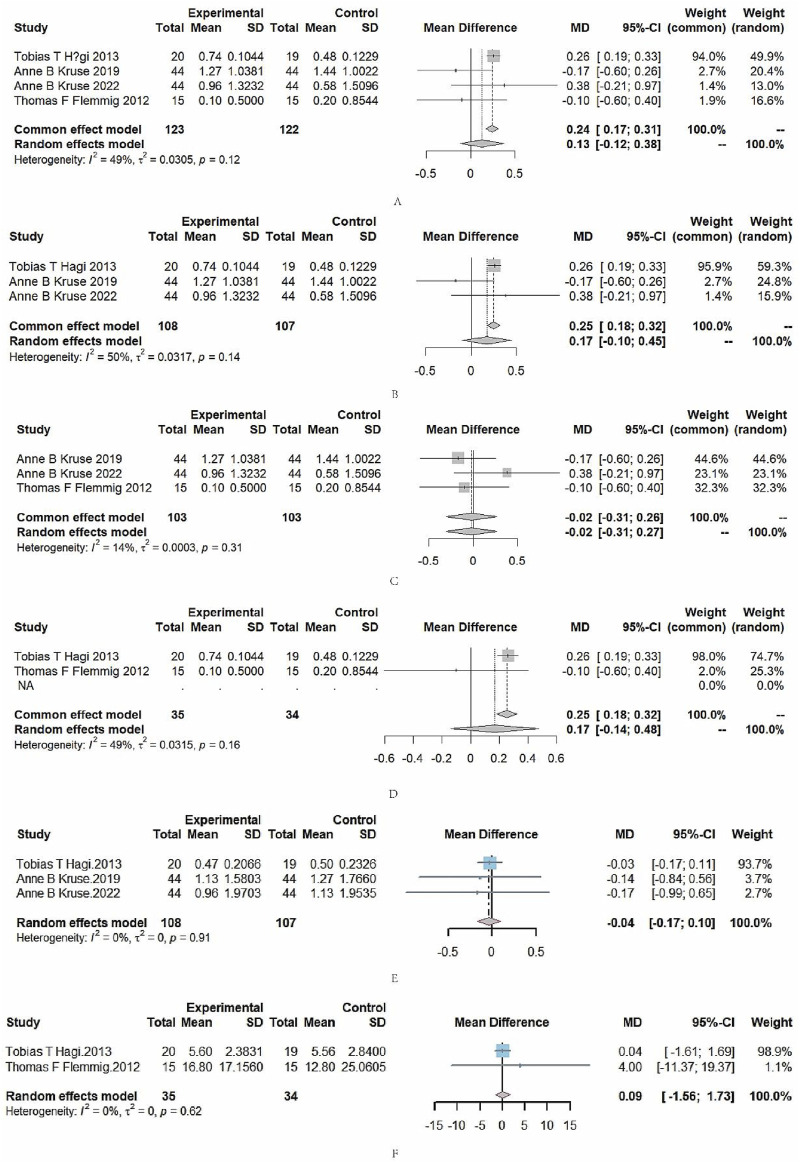
**(A)** Forest plots comparing PPD with glycine, erythritol, and trehalose. **(B)** Forest plot comparing PPD with erythritol versus trehalose. **(C)** Forest plot comparing PPD with glycine versus trehalose. **(D)** Forest plot comparing PPD with glycine versus erythritol. **(E)** Forest plot comparing CAL with trehalose versus erythritol. **(F)** Forest plot comparing BOP with glycine versus erythritol.

Since there were three kinds of subgingival air polishing powders involved, a network meta-analysis was carried out. The SUCRA was as follows: erythritol 84.1; trehalose 48.0; glycine 28.5, and the effect of reducing the PPD was ranked as follows: erythritol > trehalose > glycine. Nonetheless, the network meta-analysis showed that there was no statistically significant difference in PPD among erythritol, trehalose, glycine (Table 3). Meanwhile, as shown in Figure 4A, the PPD difference among erythritol, glycine and trehalose was not statistically significant with moderate statistical heterogeneity (I2 = 49%, P = 0.12).

**TABLE 3:** Network Meta-analysis of results PPD.

Results of network meta-analysis	Mean difference (95% CrI)	Erythritol	Glycine	Trehalose
Control	—	0.77 (0.47, 1.24)	1.13 (0.57, 2.18)	0.97 (0.59, 1.51)
Erythritol	—	—	1.46 (0.64, 3.27)	1.27 (0.64, 2.34)
Glycine	—	—	—	0.86 (0.38, 1.89)
Trehalose	—	—	—	—

Note. *PPD*, probing pocket depth; *CrI* = credible interval.

The table presents the mean differences (with 95% credible intervals) from the network meta-analysis for PPD, reduction among different interventions.

As shown in Figure 4B, the PPD difference between erythritol and trehalose was not statistically significant with no statistical heterogeneity (I^2^ = 50%, P = 0.14).

As shown in Figure 4C, the PPD difference between trehalose and glycine was not statistically significant with no statistical heterogeneity (I^2^ = 14%, P = 0.31).

As shown in Figure 4D, the PPD difference between erythritol and glycine was not statistically significant with no statistical heterogeneity (I^2^ = 49%, P = 0.16).

#### 4.2.2 CAL

As shown in Figure 4E, the CAL difference between erythritol and trehalose was not statistically significant with no statistical heterogeneity (I^2^ = 0%, P = 0.91).

Due to the lack of data related to glycine, data analysis could not be carried out, so only the data of erythritol and trehalose were compared.

#### 4.2.3 BOP

As shown in Figure 4F, the BOP difference between erythritol and glycine was not statistically significant with no statistical heterogeneity (I^2^ = 0%, P = 0.62).

Due to the lack of data related to trehalose, data analysis could not be carried out, so only the data of glycine and erythritol were compared.

## 5 Discussion

Periodontal disease is one of the most common inflammatory diseases in humans. Periodontitis remains a worldwide public health problem, with a combined prevalence of nearly 62% in adults with teeth. At the same time, periodontitis is also considered as a global public health problem, which affects not only periodontal health, but also the overall health of patients [8]. NSPT includes supragingival scaling, subgingival scaling, root planning, etc. The destruction and removal of bacterial biofilm is currently the most effective treatment for periodontal disease (Pal et al., 2021). However, these operations may have the risk of damaging the dental tissue (Bozbay et al., 2016), resulting in sensitivity to the root surface. In addition, some root surface plaque may not be cleaned due to some anatomical factors. Moreover, patient comfort is also an important factor in the evaluation of treatment methods. Many studies have shown that patients with subgingival air polishing have less pain (Kruse et al., 2018; Kruse et al., 2022), higher satisfaction, shorter treatment time, higher comfort (Mensi et al., 2021), and greater safety when compared with SRP (Martins et al., 2022).Therefore, subgingival sandblasting with low-abrasive powder has become a complementary therapy for SRP in recent years. This method takes less time and can effectively remove supragingival and subgingival biofilms, and is even expected to replace SRP (Zhu et al., 2021).

In order to solve the serious damage to the root surface and soft tissue caused by subgingival air polishing of sodium bicarbonate powder, three low-abrasive powders have been developed: glycine, erythritol, and trehalose (Alexia Vinel et al., 2022). There is currently no consensus on which subgingival air polishing powder produces the best results. In this study, meta-analysis was used to compare the efficacy of these three kinds of air-polishing powders, aiming to provide a theoretical basis for clinical decision-making.

Glycine is an amino acid and an immunomodulator of inflammatory responses in gingival tissue (Alexia Vinel et al., 2022). It is bio-compatible and highly soluble. It can remove the subgingival biofilm and clean the plaque efficiently without damaging the soft and hard tissues around the root. Glycine powder has a particle size of 25–65 μm (Alexia Vinel et al., 2022)and is a common air-polished powder at present. Studies have shown that subgingival air polishing with glycine powder can reduce the pain and discomfort of patients during periodontal treatment (Alexia Vinel et al., 2022; Bühler et al., 2015; Zhu et al., 2021), and improve the patient’s experience. Moreover, glycine sandblasting reduced the numbers of subgingival *Porphyromonas gingivalis*, *Aggregobacter actinomycetes*, and *Fusobacterium nucleatum* (Moëne et al., 2010; Zhang et al., 2021). However, some studies have shown that glycine powder enhanced the expression of pro-inflammatory genes such as TNFα, IL-8 and CCL2 by activating the NF-kB pathway, and delayed wound healing *in vitro* (Weusmann et al., 2021). *In vitro* studies have shown that glycine powder affects gingival wound healing and the viability of human gingival fibroblasts. Trehalose powder has no significant inhibitory effect on the wound healing. The effect of erythritol powder on the wound has not been reported (Wenzler et al., 2021; Weusmann et al., 2022).

Erythritol is water-soluble, chemically stable, and used worldwide as a food additive and artificial sweetener. Erythritol powder has a particle size of 14 μm (Alexia Vinel et al., 2022), which is smaller and slightly less abrasive compared to glycine powder. In addition, no adverse events were reported for GPAP (glycine air-polishing powder)and EPAP (erythritol air-polishing powder), suggesting that the powder may be a valuable alternative to glycine powder and hand devices. Meanwhile, the data suggest that EPAP may be a promising way to remove subgingival biofilm during SPT (supportive periodontal therapy) (Tobias et al., 2015).In the microbiological results, erythritol had an antimicrobial effect, via alteration of the microstructure and metabolic profile of *P. gingivalis* biofilm *in vitro* (Hashino et al., 2013; Park et al., 2018). Erythritol air-polishing powder can be successfully used in NSPT for biofilm control (Onisor et al., 2022).

Trehalose is a non-cariogenic disaccharide approved for food processing and has a similar grinding effect on dental materials as glycine (Alexia Vinel et al., 2022). Trehalose powder has a particle size similar to that of glycine, ranging from 25 to 35 μm. Both the trehalose powder and glycine powder showed statistically significant reduction in total bacterial load in subgingival air polishing, but the antibacterial effect of glycine powder was more significant (Wenzler et al., 2021).

Until now, as far as we know, there was no systematic meta-analysis of the efficacy of glycine, erythritol and trehalose subgingival air polishing powder. Our study is the first to explore this question. In this study, for PPD, although the network meta-analysis showed that there was no statistical significance in the cross-comparison of erythritol, glycine and trehalose, erythritol (SUCRA = 84.1) has an advantage over trehalose (SUCRA = 48.0) and glycine (SUCRA = 28.5) in reducing PPD. The results of direct meta-analysis showed that there was no significant statistical difference in the improvement of outcome indicators such as PPD, CAL and BOP by the three subgingival air polishing powders. In future clinical practice, the selection of the most appropriate subgingival air-polishing powder should be based on the severity of periodontal diseases, patients' oral hygiene habits, treatment tolerance, and the chemical properties of different powders. This strategy may potentially enhance therapeutic efficacy to a certain extent.

This meta-analysis has certain limitations. Due to the limited number of articles that met the inclusion criteria, only nine randomized controlled trials (RCTs) were included in this study, with a relatively small sample size of 462 patients. This made it difficult to conduct a test for publication bias, thereby limiting the generalizability of the results. Additionally, the included studies exhibited heterogeneity in experimental design, such as the implementation of interventions, follow-up duration, and patient baseline characteristics, which may have affected the comparability of the results. Regarding the inclusion criteria, due to the limited number of articles, we did not separate full-mouth and split-mouth experiments, which may have some impact on the results. A key factor in extracting valid data is the experimental observation time. In erythritol experiments, most long-term studies are conducted in stages, some of which may even last for a year. We primarily extracted data from the first stage of the experiments and selected the 3-month node. Glycine experiments were mainly short-term, lasting from 1 to 3 months. To reduce heterogeneity, we only selected data with a duration of 3 months, which is also the reason for the small amount of data. The lack of long-term follow-up data limits our assessment of long-term efficacy. In terms of data analysis, although we performed a network meta-analysis, the results showed no statistically significant differences among the three air-polishing powders (erythritol, glycine, and trehalose) in the main outcome indicators such as PPD, CAL, and BOP. Moreover, some studies lacked data on certain outcome indicators (such as BOP or CAL), preventing complete analysis. In the glycine studies, many articles mentioned the effect of subgingival air polishing on the plaque index (PI), but unfortunately, this point was rarely mentioned or data were missing in the erythritol and trehalose studies. Additionally, the criteria for the plaque index (PI) varied and were not comparable. Regarding the implementation of interventions, differences in the use of air-polishing powders across studies (such as pressure, duration, and angle of application) may have affected the consistency of the results. It is hoped that more high-quality, large-scale, prospective RCTs on subgingival air polishing will be conducted in the future to draw more reliable conclusions.

## 6 Conclusion

According to our meta-analysis, there was no statistically significant difference in the effects of glycine, trehalose, and erythritol sandblasting powder on PPD, CAL and BOP in NSPT. However, erythritol (SUCRA = 84.1) has an advantage over trehalose (SUCRA = 48.0) and glycine (SUCRA = 28.5) in reducing PPD. In the specific clinical application process, we recommend selecting the most suitable subgingival air-polishing powder based on the physical and chemical properties of the subgingival air-polishing powder, the ability to inhibit bacterial growth, the impact on wound healing, as well as the severity of the patient’s periodontal disease, oral hygiene habits, and the patient’s tolerance to treatment.

## Data Availability

The original contributions presented in the study are included in the article/supplementary material, further inquiries can be directed to the corresponding author.
